# Investigation of YAP‐1, OTX‐2, and nestin protein expressions in neuroblastoma: a preliminary study

**DOI:** 10.1002/acn3.52136

**Published:** 2024-06-26

**Authors:** Selen Kum Özşengezer, Zekiye Sultan Altun, Gamze Sanlav, Burçin Baran, Deniz Kızmazoğlu, Safiye Aktaş, Pembe Keskinoğlu, Nur Olgun

**Affiliations:** ^1^ Department of Basic Oncology Oncology Institute, Dokuz Eylül University Izmir Turkey; ^2^ Department of Clinical Oncology Institute of Oncology, Dokuz Eylül University Izmir Turkey; ^3^ Department of Pediatric Oncology Institute of Oncology, Dokuz Eylül University Izmir Turkey; ^4^ Department of Basic Medical Sciences, Department of Biostatistics and Medical Informatics Faculty of Medicine, Dokuz Eylül University Izmir Turkey

## Abstract

**Objectives:**

Neuroblastoma is the most common extracranial solid tumor in childhood. YAP (Yes‐associated protein) is a highly expressed protein in NB. Nestin is an important marker of neuronal differentiation in NB. Orthodenticle homeobox (OTX) is a transcription factor and is overexpressed in blastoma‐derived tumors. The aim of this study was to examine the potential roles of YAP‐1, Nestin, and OTX‐2 proteins in prognosis and risk stratification in neuroblastoma

**Methods:**

Tumor sections of 56 patients with different NB risk groups were analyzed. YAP‐1, Nestin, and OTX‐2 protein expression levels were evaluated by immunohistochemical staining in NB patient tissue samples.

**Results:**

YAP‐1, Nestin, and OTX‐2 protein expression levels were evaluated together with the clinical findings of NB patients. YAP‐1 was expressed in 18% of all tissues, while Nestin was expressed in 20.4%. OTX‐2 protein expression was found in 41.1% of the NB patients. YAP‐1 was expressed in 26.9% of high‐risk and 11.5% of low‐risk patients. Nestin was expressed in 24.4% high‐risk and 33.3% low‐risk patients. OTX‐2 was expressed in 68.2% high‐risk and 60% low‐risk patients.YAP‐1 was shown to provide survival advantages among risk groups.

**Interpretation:**

The findings of this study support that YAP‐1 may be a potential prognostic biomarker for staging and risk‐group assignment of NB patients. YAP‐1 expression in neuroblastoma is associated with significantly poorer survival probabilities and should be considered as a potential therapeutic target. OTX‐2 is a promising predictive biomarker candidate, but its mechanisms need further investigation in neuroblastoma, as nestin expression is not significantly linked to patient survival.

## Introduction

Neuroblastoma (NB) is the most common and deadliest extracranial solid tumor, accounting for 15% of all cancer‐related deaths in childhood.^1^ The median age of appearance of neuroblastoma is approximately 19 months. Most of the patients are diagnosed within the first 5 years.^2^ Clinically, it can occur as a primary tumor anywhere in the sympathetic nervous system that occurs in the adrenal medulla.^3^ While primary adrenal tumors may occur as a mass in the abdomen, tumors originating from sympathetic nerve roots may present with symptoms that vary according to the region of origin.^4^ Approaches to risk and treatment stratification for NB lead to mild therapy for low‐ and intermediate‐risk patients and improved outcomes for all patients. Different clinical manifestations such as spontaneous regression, metastatic and resistant disease may occur in NB. The International Neuroblastoma Staging System (INSS) has been developed to provide a common system for staging patients with NB for clinical and biological studies worldwide.^5^, ^6^ Since the aggressive surgical approach can significantly alter the INSS disease stage, an alternative approach based on clinical evaluation and imaging and independent of the extent of surgical excision has been developed.^7^, ^8^ The International Neuroblastoma Risk Group Staging System (INRGSS) staging system used in the Turkish Pediatric Oncology Group (TPOG)‐NB 2020 Protocol includes L1, L2, M, and MS stages.^2^ According to the INRG Staging System (INRGSS) criteria, locoregional tumors are categorized as L1 or L2 based on the absence or presence of tumor infiltration or invasion of nerves, vessels, and organs (image‐identified risk factors [IDRFs]).^9^ M stage is commonly used for disseminated disease. MS defines metastatic NB limited to the skin, liver, and bone marrow without cortical bone involvement in children 0 to 18 months old with primary tumors in L1 or L2.^10^

The presence of various clinicopathological and biological factors in relation to the prognosis has been analyzed in studies to date. Patients' age, tumor stage, histopathological classification, and MYCN amplification are the most frequently confirmed prognostic markers.^11^ International Neuroblastoma Pathological Classification (INPC), peripheral neuroblastic tumors (pNT); Schwannian ganglioneuroblastoma, neuroblastoma (NB, stroma‐poor); mixed (GNBi, rich in Schwannian stroma); ganglioneuroma (GN, Schwannian stroma predominant); and ganglioneuroblastoma, nodular (GNBn, composite Schwannian stroma rich/stroma‐dominant and stroma‐poor).^12^ The three classical histopathological patterns of neuroblastoma, ganglioneuroblastoma, and ganglioneuroma reflect neuronal differentiation. The fully differentiated benign response of neuroblastoma is ganglioneuroma.^13^

Useful potential prognostic markers of NB may provide clues for early diagnosis, recurrence, and treatment.^14^ NB arises from neural crest cells during embryonic development. Various signaling pathways are involved in neural crest induction.^15^ One of them is the Hippo signaling pathway. The Hippo signaling pathway is a signaling pathway that alters key target genes to control numerous biological processes, including cellular proliferation, differentiation, cell fate determination, organ size, and tissue homeostasis.^16^, ^17^ Yes‐associated protein (YAP), which is among the main components of the pathway, is a protein that plays a critical role in many types of cancer, including neuroblastoma. YAP is expressed in neural crest cells. After the maturation and differentiation of neural crest cells, the expression of YAP begins to decrease.^18^ It has been reported that YAP is highly expressed in NB and its expression levels correlate with advanced tumor staging.^19^ Data from previous studies support that YAP may be a logical therapeutic target in high‐risk NB.^20^, ^21^ Many studies emphasize that YAP is a regulator of the tumor microenvironment (TME) in NB, which affects the therapeutic response in different cancer types.^20^, ^21^, ^22^ Nuclear YAP is observed in high amounts in liver cancer with poor prognosis and also in different cancer types.^23^, ^24^ The fact that the increased amount of YAP due to tissue‐specific deletion of Mst1/2 or Lats1/2, which is one of the main components of the Hippo pathway, leads to hyperplasia, excessive organ growth, and tumor formation, supports that YAP is an oncoprotein.^23^, ^24^ On the other hand, there are many studies in the literature stating that YAP is a tumor suppressor. In one of these, YAP was found to increase the expression of proapoptotic genes by interacting with p73 in promyelocytic leukemia.^25^ In another study, anoikis was suppressed and cell migration and invasion increased by decreasing or inhibiting the level of YAP in breast cancer.^26^ A study showed in gastric cancer, YAP binds to RUNX3 instead of TEAD, the binding protein required for transcription in the nucleus, thereby reducing the tumorigenicity of MKN28 gastric cancer cells.^27^ In a recent study, YAP has been shown to be associated with cell survival, proliferation, migration, and drug resistance. It has functional importance in resistance to treatment and relapse in NB.^28^

Nestin is a cytoskeletal protein classified as an intermediate filament as well as a neuroepithelial stem cell protein identified in developing and adult brain neural stem cells (NSCs).^29^ Nestin is also expressed in various types of malignancies such as osteosarcoma, NB, glioma melanoma, and pancreatic and prostate cancers, especially in tumor vasculature.^30^ It has been reported that due to its expression in many human solid tumors, Nestin can also be used as a diagnostic and potential prognostic marker of malignancy.^30^

Orthodenticle homeobox (OTX) is a transcription factor. OTX‐2 is located in the 14q21‐22 chromosome region of human genome. It is an important gene for forebrain development and specification and retinal development.^31^ It has been reported in studies that OTX‐2 is overexpressed especially in medulloblastoma, a blastoma‐derived tumor.^31^, ^32^ The gain or loss of OTX genes promotes tumorigenesis as a result of their abnormal/defective effects on growth and differentiation.^33^ Deregulated expression of OTX genes has been described in leukemia and lymphomas, and many solid tumors such as medulloblastomas, aggressive Non‐Hodgkin lymphomas, breast carcinomas, colorectal cancers, and retinoblastoma.^34^ However, studies to date have not revealed whether YAP‐1, Nestin, and OTX‐2 have a decisive role in NB, especially in terms of risk group stratification. In particular, the mechanisms of action of YAP‐1 and OTX‐2 on NB have not been fully elucidated so far. This study suggests that they may be new descriptive biomarkers for possible treatment targets. In this study, it was aimed to reveal the potential determinative roles of tumor suppressor/oncogene YAP‐1, stem cell markers Nestin, and transcription factor OTX‐2 in NB, especially in terms of prognosis and risk groups.

## Material and Methods

### Experimental groups

Permission for this research study was obtained from the Non‐Interventional Research Ethics Committee of Dokuz Eylül University (Decision No: 2022/16–03). Consent forms were obtained from the children's families during the sampling and storage stages. The study consisted of biopsy samples from 56 patients (30 females and 26males). All tissues examined were pretreatment samples. Low‐risk group in NB was defined as Stage 1 and Stage 2A at all ages, and as Stage 1, 2, 3, or 4S if <18 months. Medium‐risk group was defined as Stage 2 and Stage 3 at all ages, and Stage 4 if <18 months. The high‐risk group was defined as >18 months and Stage 4 tumors.

MYCN amplification was accepted as positive for samples with RT‐PCR values greater than 10‐fold amplification and negative for less than 10‐fold amplifications. Within the scope of TPOG, samples from different stages of NB formalin‐fixed paraffin‐embedded (FFPE) tissues of patients in Dokuz Eylül University, Department of Pediatric Oncology and Basic Oncology archive were randomly selected. Sections were taken from paraffin blocks to adhesive slides, and staining protocols were applied. The patients were classified according to the TPOG‐2009 protocol as 26 high risk, 12 intermediate risk, and 18 low risk.^35^

### Antibodies and samples

In this study, polyclonal human YAP‐1 (Thermo‐86396), OTX (Thermo‐85774), and Nestin (BiossUSA‐ bs‐0008R) and secondary (Ventana 760–4311) antibodies were used. Antibodies were stored in accordance with the manufacturer's instructions, and dilution rates were taken into account. The companies have cited the literature for the dilution rate of each antibody and set out the specific ranges in the product data sheets. By performing the control staining, the dilution rates of the antibodies were optimized for YAP‐1 (1:100), OTX‐2 (1:200), and Nestin (1:200), respectively. Paraffin blocks were selected from NB patient tissues to include low‐, medium‐, and high‐risk groups.

### Immunohistochemistry staining

Tissue slides were kept overnight in an oven at 60°C before staining.^36^ Slides removed from the oven were kept in xylol for 1 hour for deparaffinization. It was then passed through a decreasing series of alcohol. Antigen retrieval was performed by boiling slides in citrate buffer in the microwave. After applying hydrogen peroxide and washing steps, human‐reactive primary IgG antibodies were applied (an optimization study was done beforehand). Then, multimer HRP secondary antibody was dripped onto the slides and incubated. In the final step, the slides were catalyzed by applying DAB with hydrogen peroxide.

Then, nucleus was counterstained with hematoxylin; slides were transferred through increasing alcohol series and taken into xylene for the clearing step. It was then visualized under a light microscope (Olympus BX50). YAP‐1 and Nestin proteins showed nuclear localization, while OTX‐2 showed both nuclear and cytoplasmic localization.^37^, ^38^, ^39^

Appraisal of the immunohistochemical YAP‐1, Nestin, and OTX‐2 expression was based on a semiquantitative microscopy‐based scoring system. Protein expressions were explained as percentages by scanning 10 different areas in each tumor tissue and calculating the average value. Relationships between protein expressions and patients' survival, event, risk, and MYCN status were evaluated. Immunohistochemically stained sections were evaluated for staining quality and histochemistry by a pathologist involved in the study.

### N‐MYC determination with RT‐PCR

DNA isolation was performed from NB paraffin tumor tissue samples. Concentrations of isolated DNA samples were measured fluorimetrically with the Quibit device. These samples were evaluated for N.MYCN amplification and 11q23 deletion by real‐time PCR reaction. The evaluation was performed by calculating the target and reference CT values of control DNA and patient samples. Primer and enzyme mixtures containing specially designed labeled probes for these regions were used to detect gains or losses in these regions. For MYCN simultaneous PCR, an enzyme mixture containing the primer pair 5′‐GTGCTCTTCCAATTCTCGCCT‐3′ and 5′‐GATGGCCTAGAGGAGGGCT‐3′ and the 5′‐FAMCACTAAAGTTCCTTCCACCCTCTCCT‐TAMRA‐3′ Taqman probe designed specifically for MYCN gene was used. For 11q23 deletion analysis, primer pair 5′‐ATCTGGAGGCAGCACAGCT‐3′ and 5′‐TACACTGGATTATACCCTGGCTGG‐3′ and 5′‐FAM‐CCATGATCACAGAGACCATCATTGAAA‐BBQ(tamra) designed specifically for ARCN1 (Archain 1) gene located in 11q23.3 region. The enzyme mixture containing the Taqman probe was used. All PCR reactions were prepared in eight PCR tubes and performed using Nano Real‐time PCR device (Roche), and relative quantitation was performed for analysis. Relative quantitation was performed using the delta, delta cycle threshold (Δ/ΔCT) analysis method. Healthy reference DNA (Std DNA) was used as the calibrator. These results were confirmed in another quantitation method, absolute quantitation, again using reference DNA standards.

### Statistical analysis

The Fisher exact test was used to assess the relationship between categorical outcomes (risk group, MYCN amplification, and survival status) and categorical variables (YAP‐1, Nestin, and OTX‐2 expression patterns). To ascertain whether there is a difference between the groups, the independent samples *t*‐test was used to assess the mean EFS and OS time. The expression of data is Mean ± SD (n). Events included relapse, secondary cancer, and death from any cause in the EFS analysis. The period between the diagnosis and the first event, or the final patient contact in the case of no event, was used to compute the “time to event.” The time from enrollment to death, or the patient's final contact if they were still alive, was used as the time to event for OS analysis. The survival curves were created using the Kaplan–Meier method, and a log‐rank test was employed to compare the curves. Before the start of this study, the disease stage and risk classification systems were established. These systems use the patient's age at diagnosis, the tumor's histology, and its molecular characteristics to classify patients. Confounding variables include stage and risk categorization when comparisons are performed over the whole cohort. Therefore, to better support our results we performed a multivariate regression survival analysis with the previously mentioned confounding factors.

All statistical analyses were performed using IBM SPSS Statistics Version 29 (IBM, USA), and *p* < 0.05 was considered significant.

## Results

### Patient characteristics

In total, 56 NB patient tissues were included in this study. The gender consisted of 30 female and 26 male patients. The ages of the patients ranged from 1 month to 11 years. Patients were staged according to the criteria defined by the Turkish Pediatric Oncology Group‐2020 and the INSS. Risk groups were determined according to the International Neuroblastoma Risk Group Staging System (INRGSS) classification system. Of the patients, 18 were classified as low risk, 12 as medium risk, and 26 as high risk. Considering the stages of the disease, 12 patients were at Stage I, 5 patients at Stage II, 8 patients at Stage III, and 31 patients at Stage IV. Due to the low number of patients in the risk groups, protein expressions were expressed as the low‐risk group by combining the data of low‐risk and intermediate‐risk patients (Table 1).

**Table 1:** Neuroblastoma patient characteristics.

Characteristic	Total (*n* = 56)
Age (months)	Mean ± SD
	37.95 ± 42.12 (*n* = 56)
Gender	*n* (%)
Female	30 (53.6%)
Male	26 (46.4%)
Risk group	*n* (%)
Low	18 (32.1%)
Medium	12 (21.4%)
High	26 (46.4%)
Stage	*n* (%)
I	12 (21.4%)
II	5 (8.9%)
III	8 (14.3%)
IV	31 (55.4%)
MYCN Status	*n* (%)
Positive	12 (21.4%)
Negative	44 (78.6%)
Survival status	*n* (%)
Alive	45 (80.4%)
Dead	11 (19.6%)
EFS (months)	Mean ± SD
	37.01 ± 28.97 (*n* = 56)
OS (months)	Mean ± SD
	40.29 ± 27.49 (*n* = 56)

The characteristic features of the 56 patients participating in the study were staged according to the criteria determined by the Turkish Pediatric Oncology Group‐2020 and INSS, and the risk groups were determined according to the International Neuroblastoma Risk Group Staging System (INRGSS) classification system.

### The expression of YAP‐1 is significant with EFS and OS in high‐ and low‐risk groups in NB

**Figure 1:**
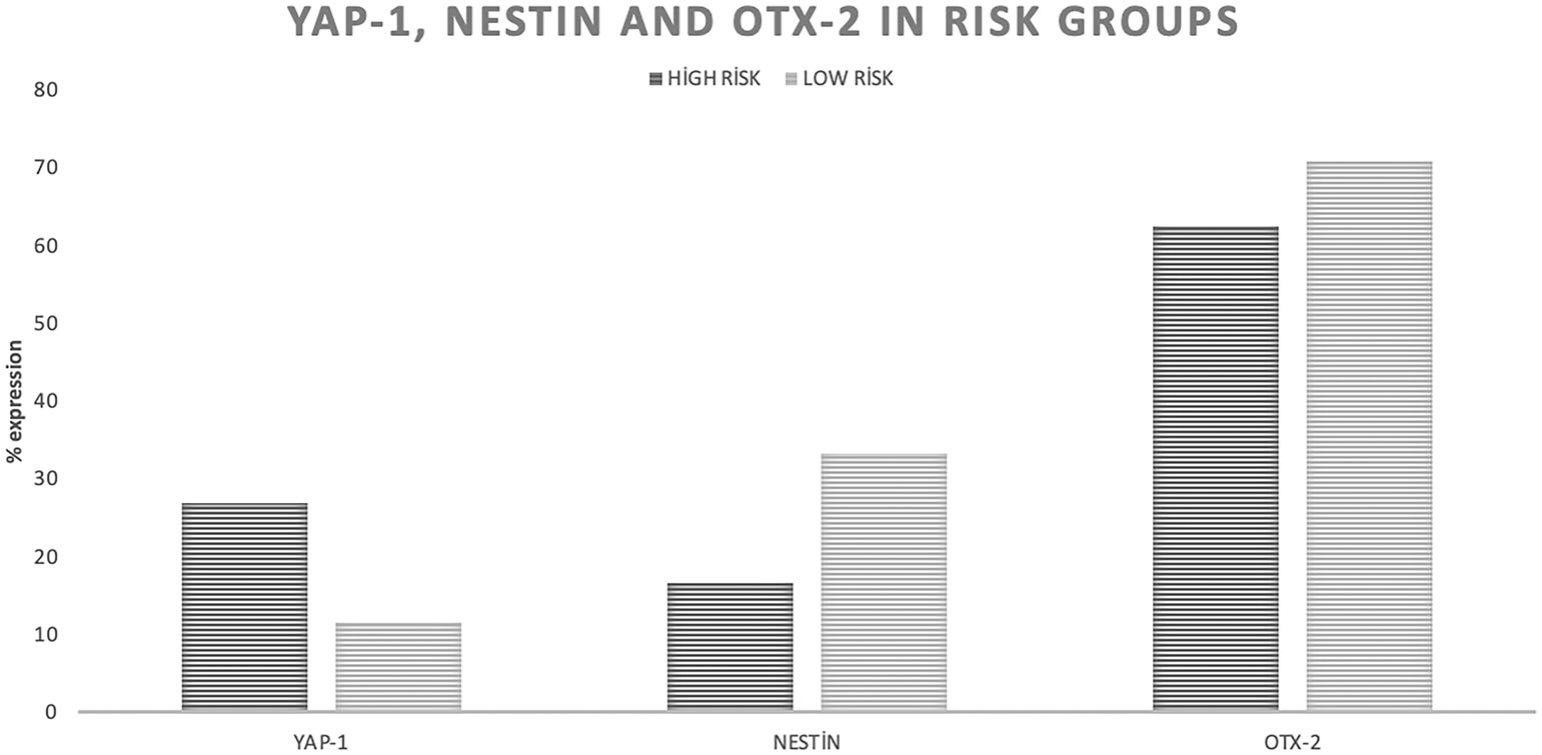
Comparison of YAP‐1, Nestin, and OTX‐2 protein expressions according to NB risk groups. Evaluation of the expression of YAP‐1, NESTIN, and OTX‐2 in neuroblastoma according to risk groups is shown.

**Figure 2:**
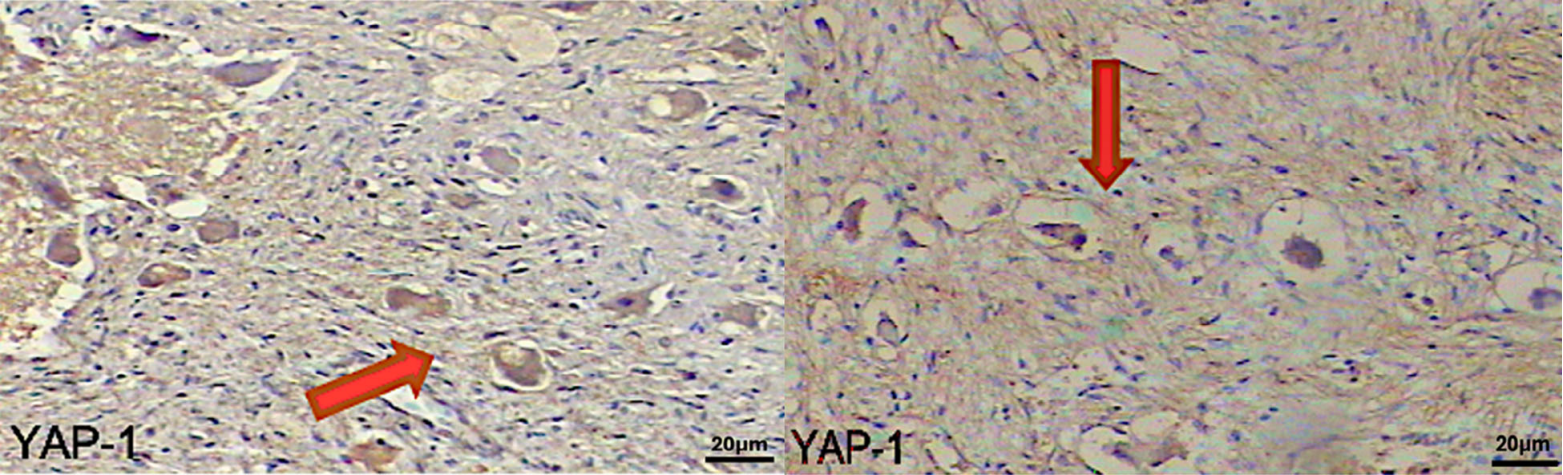
YAP‐1 protein expressions in MYCN‐negative and MYCN‐positive NB cases. Strong of YAP‐1 staining is demonstrated in neuroblastoma patient samples. The positive nuclear staining shown red arrows (20X). A 1:100 dilution was applied for YAP‐1.

**Figure 3:**
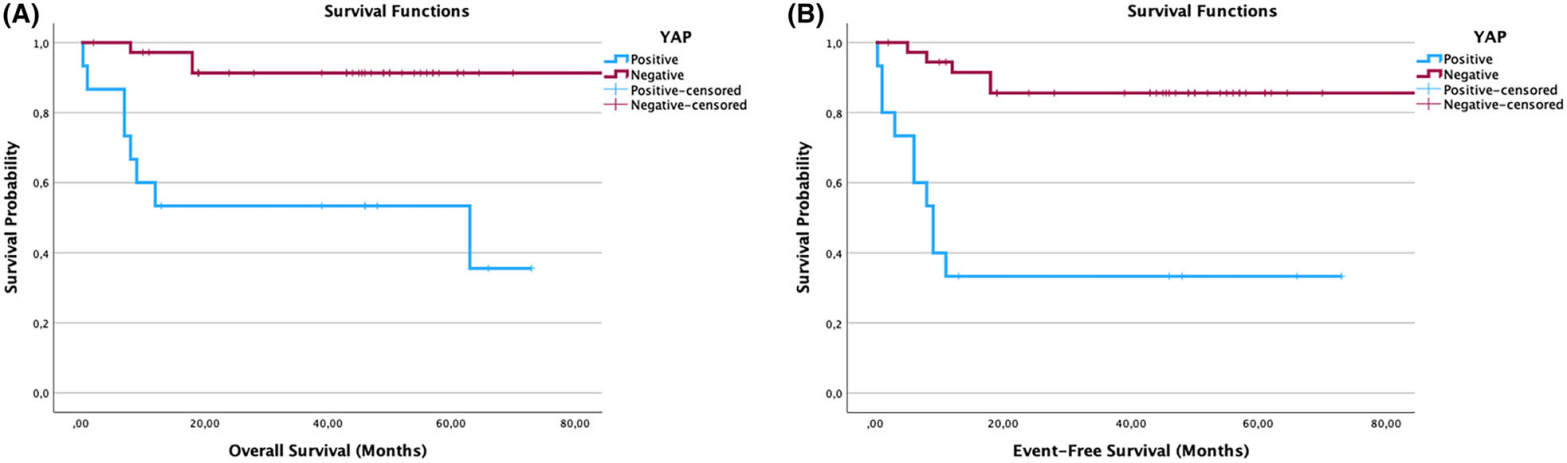
YAP‐1 case graphs for neuroblastoma (NB) showing overall survival (OS) and event‐free survival (EFS). YAP‐1 positive versus negative case graphs for overall survival (OS) and event‐free survival (EFS) in neuroblastoma (NB) patients. The Kaplan–Meier curves showing YAP‐1 positive versus negative NB cases. OS (A) and EFS (B) of NB patients with YAP‐1 negative (red line) and YAP‐1 positive (blue line) tumors. Log‐rank test was used to compare YAP‐1 positive and negative NB cases.

In the study, YAP‐1 was found to be stained more strongly, especially in ganglioneurons (Figs. 1 and 2). YAP‐1 expression level was determined in 18% of all patients. When evaluated according to the stage of the disease, expression was 27.3% in Stage I, 12.5% in Stage III, and 21.4% in Stage IV. Expression was not detected in stage II. When YAP‐1 expression levels were evaluated according to risk groups, it was determined that it was expressed in 26.9% of high‐risk and 11.5% of low‐risk patients (Fig. 1). YAP‐1 showed strongly significant results in OS and EFS rates of NB patients. When OS and EFS rates were compared between NB patients with YAP‐1 positive and negative tumors using a Log‐rank survival analysis, NB patients with YAP‐1 positive tumors were shown to have significantly lower EFS and OS rates (*p* < 0.001) (Fig. 3).

### YAP‐1, OTX‐2, and Nestin protein expressions are higher in MYCN+ NB patients

**Figure 4:**
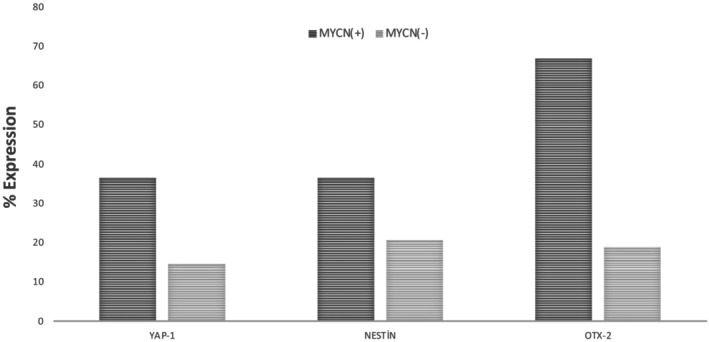
Expression percentages of YAP‐1, OTX‐2, and Nestin according to MYCN status in NB patients. Evaluation of the expression of YAP‐1, NESTIN, and OTX‐2 in neuroblastoma according to MYCN(+) and MYCN(−) is shown.

**Figure 5:**
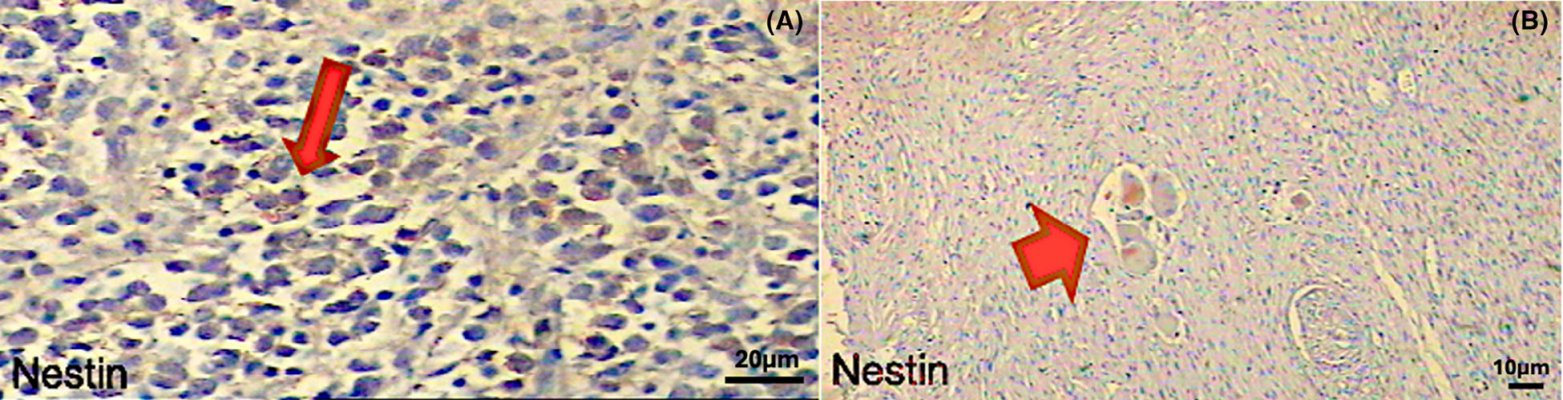
Nestin immunohistochemical staining. Samples from patients with neuroblastoma show strong Nestin staining. (A) The positive nuclear staining was indicated by red arrows (20X). (B) Red arrowheads denote positive nuclear staining (10X). For Nestin, a 1:200 dilution was used.

YAP‐1, OTX‐2, and Nestin protein expression levels were evaluated according to the patients' MYCN expression status. The YAP‐1 protein was found to be expressed in 36.4% of MYCN (+) patients, while it was positively expressed in 14.6% of MYCN (−) patients (Fig. 5). While the Nestin protein was expressed in 36.4% of MYCN (+) patients, it showed an expression of 20.6% of MYCN (−) patients. OTX‐2 protein expression was seen in 66.7% of MYCN (+) patients, while it was expressed in 18.8% of MYCN (−) patients (Fig. 4).

### Analysis of Nestin and OTX‐2 expression

**Figure 6:**
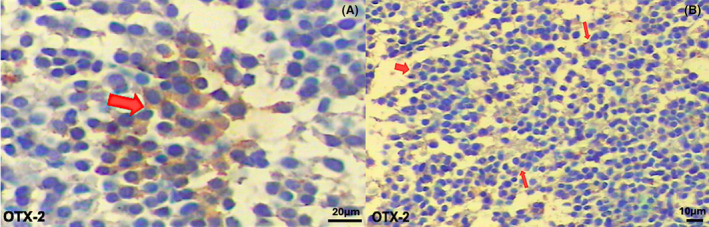
OTX‐2 immunohistochemical staining. Neuroblastoma patient samples have highly positive nuclear and cytoplasmic staining cells (OTX‐2 staining). (A) Red arrows showed positive staining of the cytoplasm and nucleus (20X). (B) Positive cytoplasmic and nuclear staining is indicated by red arrowheads (10X). OTX‐2 was diluted at a ratio of 1:200.

**Figure 7:**
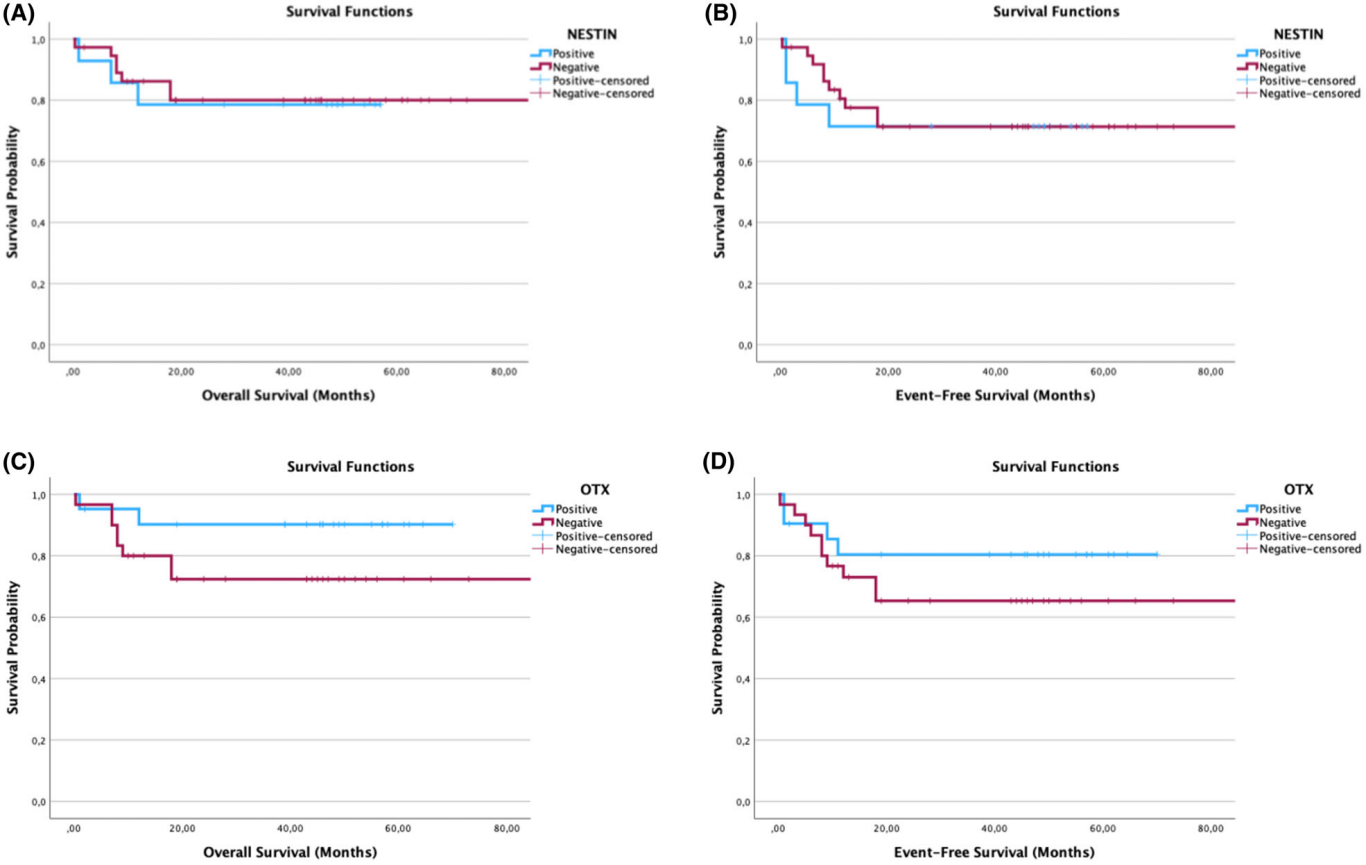
OTX‐2 and Nestin case graphs for neuroblastoma (NB) showing overall survival (OS) and event‐free survival (EFS). OTX‐2 and Nestin positive versus negative case graphs for overall survival (OS) and event‐free survival (EFS) in neuroblastoma (NB) patients. The Kaplan–Meier curves showing OTX‐2 and Nestin positive versus negative NB cases. OS (A) and EFS (B) of NB patients with OTX‐2 and Nestin negative (red line) and OTX‐2 and Nestin positive (blue line) tumors. Log‐rank test was used to compare OTX‐2 and Nestin positive and negative NB cases.

Nestin was found to be expressed in a total of 20.4% of all patients. Nestin expression was 33.3% in Stage I, 25% in Stages II and III, and 20.8% in Stage IV. Nestin was expressed in 24.4% high‐risk and 33.3% low‐risk patients (Fig. 1). When the relationships between nestin expression and survival, event status, and risk groups in NB were evaluated, no statistical difference was found (*p* > 0.05, chi‐square test). Unfortunately, Nestin did not show significant results in OS and EFS rates of NB patients. Nestin was found to stain more strongly, especially in the nuclear of ganglion neurons (Figs. 5, 6, 7).

OTX‐2 protein expression was found to be 41.1% in total NB patient tissues. According to the stage of the disease, OTX‐2 expression was 54.5% in Stage I, 75% in Stage II, 83.3% in Stage III, and 66.7% in Stage IV. As the expression level was evaluated according to risk groups, it showed expression in 68.2% high‐risk and 60% low‐risk patients (Fig. 1). The EFS and OS rates of OTX‐2 positive and negative tumors showed statistically similar results. No statistically significant difference was found in the survival analysis (*p* > 0.05). OTX‐2 was found to be stained more strongly in the nuclear and cytoplasmic of ganglion neurons (Figs. 6 and 7).

### Multivariate survival analysis indicates YAP‐1 and OTX‐2 expressions are significantly associated with NB survival

The disease stage and risk classification systems were set up before the commencement of this study. These approaches categorize patients based on the tumor's molecular features, histology, and age at diagnosis. As a result, risk assessment and disease stage become confounding variables. Therefore, we conducted a multivariate Cox regression survival analysis including the previously indicated confounding factors to further validate our findings. Variables that show *p* values <0.1 as a result of univariate survival analysis and confounding factors such as age at diagnosis were included in Model 1 which consists of five independent variables as shown in Table 2. Risk classification was not included in Model 1 to avoid multicollinearity. Two independent variables which did not show significant results in Model 1 were excluded, and another model was performed with the remaining three independent variables as shown below in Model 2 (Table 2).

**Table 2:** Modeling of the results of Cox regression.

	Model 1				Model 2			
	Exp (*B*)	95.0% CI for Exp (*B*)		Sig.	Exp (*B*)	95.0% CI for Exp (*B*)		Sig.
		Lower	Upper			Lower	Upper	
YAP‐1	14.789	3.204	68.275	**0.001**	13.065	2.941	58.044	**0.001**
OTX‐2	6.083	1.097	33.717	**0.039**	5.817	1.064	31.809	**0.042**
MYCN	2.926	0.720	11.883	0.133	3.869	0.959	15.610	0.057
AGE	0.989	0.968	1.010	0.307				
STAGE	1.652	0.392	6.960	0.494				

There are five independent variables in Model 1. Model 1's nonsignificant results for two of the independent variables were eliminated, and Model 2's remaining three independent variables were used to form a new model. The values obtained are statistically significant since *p*< 0.05.

As a result of Cox regression analysis using Model 2, YAP‐1 retained to show significant results in survival in multivariate survival analysis as it also showed significant results in univariate survival analysis. On the other hand, OTX‐2 did not show significant results in univariate survival analysis; however, cox regression model showed that OTX‐2 is significantly associated with higher EFS and OS rates of NB patients.

## Discussion

Thanks to extensive research into neuroblastoma, the prognosis for patients has improved somewhat in recent years, with the 5‐year survival rate increasing from 52% to 74%. However, this is associated with higher cure rates for those suffering from low‐risk neuroblastoma. However, the percentage of cures for patients with high‐risk neuroblastoma has not changed significantly. Therefore, it is crucial to understand the molecular features and genetic variations associated with the pathophysiology of neuroblastoma in order to design tools for timely diagnosis and targeted therapy.^27^ Over the last decade, researchers have focused on developing new tools to help clinicians guide NB patients through risk assessment and survival prediction. Some researchers have investigated single‐nucleotide polymorphisms (SNPs) on different genes that regulate NB prognosis.^27^ Numerous SNPs linked to neuroblastoma risk have been found through genome wide association studies (GWAS). The ALKBH5 gene, linked to NB susceptibility, has been reported to inhibit NB progression by reducing YAP expression and activity, and to promote malignant progression by inhibiting SPP1 expression in high‐grade neuroblastoma, where it is often amplified and upregulated.^27^ Another biomarker study evaluated the potential of alternative splicing of TME‐related genes as predictive biomarkers.^40^ Tang et al. described an important step in the evolution of NB caused by PTBP2‐induced monocytes/Mφs and showed that PTBP2‐mediated RNA splicing promotes immunological compartmentalization between NB cells and monocytes. The clinical and biological importance of PTBP2 in the development of NB is further revealed by this study, which suggests that RNA splicing triggered by PTBP2 promotes immunological compartmentalization and is harbinger of a favorable outcome in mediastinal NB.^40^

N‐MYC expression is the most clinically important prognostic risk factor in NB. Currently, survival in advanced disease is at most around 50%. Due to the heterogeneous nature of NB, new biomarkers are still needed for the diagnosis, prognosis, and monitoring of treatment response. In this study, we evaluated whether YAP‐1, OTX‐2, and Nestin, which may be important for NB, could be new descriptive markers for the determination of stage and treatment strategies, especially according to risk groups and N‐MYC expression status. In adults, OTX is expressed in sensory organs and is involved in many processes such as the formation of the ciliary body and inner ear morphogenesis. Due to the critical role of OTX, mutations in these genes lead to inborn somatic or metabolic defects in humans. In particular, the gain or loss of OTX genes leads to tumorigenesis through errors in growth and differentiation.^33^ OTX‐1 and OTX‐2, which are homeobox genes involved in the development and differentiation of neuronal cells, are expressed early in the proliferating cell layers of the human fetal brain. OTX‐1 is expressed mainly in the neocortex, while OTX‐2 is expressed in the archicortex, diencephalon, rostral brain stem, and cerebellum.^41^ Leukemia, lymphomas, and other solid tumors, including medulloblastomas, aggressive non‐Hodgkin lymphomas, breast carcinomas, colorectal malignancies, and retinoblastomas, have all been linked to the dysregulated expression of OTX genes.^33^ Both normal sinonasal mucosa and tumors have been observed to differantially express OTX‐1 and OTX‐2. Interestingly, only OTX‐1 was identified in non‐intestinal type adenocarcinomas, while OTX‐2 was selectively expressed only in olfactory neuroblastomas. Interestingly, no OTX gene was expressed in sinonasal intestine‐type adenocarcinomas. In conclusion, it is possible that the OTX‐1 and OTX‐2 genes contribute to the pathophysiology of several sinonasal neoplasm types.^33^ In a study examining whether there is a correlation between gene expression and histopathologic and clinical features, it was reported that when the frequency of OTX‐1 and OTX‐2 gene expression was examined in 60 medulloblastoma samples, it was found that OTX‐1 gene was expressed in 52% of the cases. Expression varied by histologic type (desmoplastic), location (mostly hemisphere), and age (more in adults). In 62% of cases, OTX‐2 gene expression varied according to histologic type (classical and anaplastic), location (mostly vermis), and age (more in younger age groups). Leptomeningeal metastasis development was found to be statistically correlated with OTX‐2 gene expression.^42^ In another study, OTX‐2 overexpression was detected in 86% (19/22) of the main adult retinoblastoma tumors examined. Levels of pRB in retinoblastoma were shown to be elevated by OTX‐2 inhibition.^43^ OTX‐2 has been shown in another investigation to maintain stemness in Group 3 and 4 medulloblastoma subtypes. As a transcription factor, OTX‐2 preferentially activates and represses genes that favor the development of aggressive forms of medulloblastoma. OTX‐2 exhibits a strong propensity to bind in a complex with several other important oncogenes, MYC being one of the most significant.^44^ In the case of medulloblastoma, OTX‐2 and MYC were shown to bind tightly to each other in the transcription start sites of genes that contribute to the cancer phenotype. To further strengthen this link, Adamson et al. used PCR and ChIP assays to show that endogenous OTX‐2 binds to the MYC promoter region. Downregulation of MYC was achieved by knockdown of OTX‐2 with OTX2‐specific siRNA.^45^ To date, there is no research on OTX‐2 expression analysis in NB patient tissue in the literature. However, one study showed that OTX2 expression in gliomas was absent in five of twelve patients, low expression in six, and moderate expression in one.^39^ In this study, we examined OTX‐2 expression in NB patient tissues and its correlation with survival for the first time. One study reported that overexpression of OTX‐1 is associated with tumor‐promoting effects in various malignancies.^46^ The study showed that OTX1 expression level is an individual prognostic factor for esophageal squamous cell carcinoma (ESCC) patients when OTX is overexpressed in ESCC tissues compared to normal tissues.^46^ OTX‐2 has been shown to be a very crucial target of N‐MYC during inner ear development and that N‐MYC can directly regulate OTX‐2.^47^ In a retinoblastoma study, loss of OTX‐2 expression also causes a decrease in C‐MYC expression, one of the genes involved in retinoblastoma tumorigenesis.^47^ In this study, for the first time, whether OTX‐2 expression can be a prognostic marker for NB was evaluated in patient tissue samples according to risk groups. OTX‐2 expression was not significantly different in NB risk groups. However, OTX‐2 protein expression was detected in 66.7% of MYCN (+) patients and showed a significantly increased expression pattern compared to MYCN (−) patients. In this respect, it can be evaluated whether the presence of OTX‐2 expression in MYCN‐positive patients and its examination in larger sample series according to risk groups will be important in terms of prognosis and treatment.

OTX‐2 did not show significant results in univariate analyses and showed significant results in the multivariate regression model which consists of three independent variables including a well‐known prognostic factor MYCN. This variation could be due to the regulatory role of MYCN on OTX‐2 expression, raising the question of the possible involvement of an OTX2‐MYC interaction in NB development. To date only one study was published investigating a relation between OTX‐2 and MYCN. The study by Vendrell et al. suggested that OTX‐2 may be regulated by MYCN in the mammalian cochlea and study results showed that OTX‐2 is a downstream target of MYCN and acts as a developmental suppressor.^48^ Another study revealed that OTX2 works synergistically with MYC in oncogenesis in medulloblastoma.^35^ Due to its function as a transcription factor, OTX2 activates and downregulates genes that help the aggressive medulloblastoma subtypes develop. OTX2 exhibits a strong affinity for forming complexes with numerous other significant oncogenes, with MYCN being one of the most significant among them.^49^ Gene expression in the medulloblastoma was found to be upregulated by OTX2 and MYC binding in close proximity to each other at transcription start sites of genes contributing to the cancer phenotype. Adamson et al further validated this relationship, confirming that OTX2 binds to the promoter region of MYCN. OTX2 silencing caused the downregulation of MYCN.^36^ The association between OTX2 and MYCN at the transcriptional level in other malignancies may also be the reason behind our interesting finding regulating NB prognosis.

YAP, a transcriptional co‐activator, interacts with the TEAD family of transcription factors to initiate the transcription of downstream target genes important for the growth and development of organs. TAZ, a paralog of YAP, is a transcriptional co‐activator with a PDZ‐binding motif. YAP has been shown to have a role in cell identity and tumor initiation, metastasis, angiogenesis, and resistance to chemotherapy in a variety of solid tumors, including head and neck, lung, colon, pancreas, and ovary. YAP has been linked to rhabdomyosarcoma, osteosarcoma, Ewing sarcoma, and neuroblastoma, among other malignancies that affect children and young adults.^20^ The mechanism of action of YAP‐1 protein has not yet been fully explained in NB. In studies conducted to date, both *in‐vitro* and *in‐vivo* expression levels of YAP‐1 have been evaluated.^27^ In one study, YAP‐1 expression correlated with advanced tumor stage, especially in cisplatin‐resistant NBs, and YAP‐1 was shown to support tumorigenesis and invasion.^27^ In the same study, evaluation of the relationship between YAP inhibition, tumor growth, and cisplatin resistance reveals YAP is a potential therapeutic target for cisplatin‐resistant NB.^27^ In another study using Gene Expression Profiling Interactive Analysis (GEPIA) databases, it was possible to determine that YAP‐1 expression was significantly higher in cholangial carcinoma (CHOL), lymphoid neoplasm diffuse large B‐cell lymphoma (DLBC), glioblastoma multiforme (GBM), pancreatic adenocarcinoma (PAAD), STAD, and thymoma (THYM) tumor tissues compared to normal controls. On the other hand, YAP‐1 expression was considerably reduced in tumor tissues compared to normal control tissues in cases of uterine corpus endometrial carcinoma (UCEC), bladder urothelial carcinoma (BLCA), paraganglioma (PCPG), adrenocortical carcinoma (ACC), and uterine carcinosarcoma (UCS).^40^ YAP‐1 expression has been used as an inclusion criterion in two open and one closed clinical trials involving neuroblastoma of the central nervous system. One study is in Phase 1 (open), and one is in Phase 4 (open), these studies use YAP‐1 expression and central nervous system neuroblastoma as an inclusion criterion.^41^ In a study, YAP‐1 expression was investigated together with many genes in a retrospective cohort of 46 primary pediatric neuroblastoma patients, 30 of which were treatment‐refractory and 16 of which were after chemotherapy. According to the INPC categorization, YAP‐1 expression was much greater in tumors that were differentiating compared to poorly differentiated tumors in terms of connection with the differentiation. In summary, neuroendocrine lineage transcription patterns are highly predictive of prognosis in neuroblastoma, and YAP‐1 evaluation may contribute to a more accurate characterization of recurrence risk in neuroblastoma patients.^42^ The impact of YAP‐1 on glioma prognosis was examined in a different investigation. YAP‐1 was highly expressed in 62 (31.0%) and lowly expressed in 138 (69.0%) of the 200 gliomas in a tissue microarray.^43^ In this study, the expression of YAP‐1 was found to be higher in high‐risk patients, but it was also observed in low‐risk patients' tissues to a lesser extent. It was determined that YAP‐1 expression was increased in MYCN‐positive NB cases compared to MYCN‐negative ones. The considerably better survival rates of NB patients with YAP‐1 positive tumors compared to patients with YAP‐1 negative tumors in both high‐risk and low‐risk groups were another encouraging result of this study. In this regard, considering the significant survival results in the study group, the increase in YAP‐1 expression can be considered as a prognostic biomarker that leads to a significantly worse prognosis in NB patients.

Nestin is an intermediate filament protein located in the cytoplasm of most brain cancer cells, although it has been detected in the nuclei of human neuroblastoma and medulloblastoma cell lines. These data suggest that Nestin can bind directly to DNA or intranuclear proteins.^44^ Although both low‐grade and high‐grade tumors were shown to express Nestin in a different study, the majority of gliomas with high levels of Nestin expression were high‐grade gliomas, including glioblastomas, anaplastic oligodendrogliomas, anaplastic astrocytomas, and anaplastic oligoastrocytomas.^45^ A retrospective study by Behling et al. on a sample of 113 patients with glioblastoma (GBM) patients found that Nestin did not independently affect prognosis and did not differ by age or clinical status.^39^ In another study, a retrospective analysis of 95 GBM patients who underwent primary surgical resection, Matsumoto et al. found that Nestin was largely not overexpressed in GBM tissues in lesions with pseudopalisading characteristics (Ps), but in non‐Ps or non‐peri‐necrotic lesions.^46^ The Radiation Therapy Oncology Group (RTOG) evaluated the prognostic significance of Nestin expression in newly diagnosed GBM patients treated in prior prospective clinical trials, given the variable expression of Nestin in GBM observed in another study and its possible role as a marker for a dedifferentiated and potentially it aimed to determine whether there is a more aggressive phenotype.^47^ The findings provided do not support the predictive significance of Nestin expression in newly diagnosed GBM, despite the fascinating association between it and the histological grade of gliomas. Therefore, additional studies evaluating Nestin expression are needed.^47^ A small‐scale investigation of Nestin expression in non‐small‐cell lung cancer (NSCLC) was conducted in a different research. Studies were conducted to determine the predictive importance of Nestin expression for survival in resected NSCLC patients as well as the connection between it and clinicopathological characteristics. It was stated that Nestin expression in tumor samples in 27 out of the 171 patients with NSCLC (15.8%).^50^ The results of Ryuge's study suggest that Nestin expression may be utilized as a possible marker to identify individuals who should receive adjuvant chemotherapy and is a prognostic indicator of poorer survival in resected NSCLC patients.^50^ In a study, where Nestin expression was evaluated immunohistochemically in cases of nephroma, rhabdomyosarcoma, NB, rhabdoid tumor, and desmoplastic small round cell tumor, it was shown to be largely positive.^51^ MYCN protein has also been proposed as a transcriptional activator of Nestin in NB. Again, since NB frequently amplifies the MYCN gene, resulting in increased mRNA levels, it was predicted that amplified cases would show higher Nestin expression than nonamplified cases. However, no relationship was found between Nestin staining and MYCN gene copy number.^27^ In this study, Nestin expression was found to be higher in MYCN (+) patients than in MYCN (−) patients, consistent with the literature. However, the results of this study do not support the hypothesis that Nestin can be a predictive biomarker in NB. No statistically significant relation was found between Nestin expression in NB tissues and the patient's survival. Although recent research has attempted to elucidate the initiation mechanisms of NB, we still have limited knowledge about the biology of NB. Limitations of this study include the inability to obtain sufficient patient samples. On the other hand, to control the survival and quality of life of NB patients, early detection or prediction of the disease following low‐toxic treatments, including molecular targeted drugs, is required.^52^

## Conclusion

This study demonstrated that YAP‐1 can significantly outperform existing risk variables in predicting neuroblastoma patient survival expectancy. Furthermore, the most important result of this study is the demonstration that there is a significant difference in survival in NB linked to YAP‐1 protein expression patterns. Since our results showed relatively significant survival results, OTX‐2 may also be a predictive biomarker in neuroblastoma, but this protein needs to be further investigated in a larger sample size. In this study, increased YAP‐1 and OTX‐2 protein expressions in MYCN‐positive patients compared to negative patients suggest that evaluating these protein expressions in terms of the prognosis of NB patients may be a useful approach. These two molecules (YAP‐1 and OTX‐2), which have the potential to shed light on the literature, need to be examined for their yet undetermined mechanisms for NB. To elucidate the roles of OTX‐2 and YAP‐1, in NB, they should be investigated in further mechanistic and *in‐vivo* experimental models, and the results should be confirmed in clinical samples with increased sample size.

## Author Contributions

All authors participated in the design and conceptualisation of the manuscript. D.K. and N.O. provided the samples for the study. S.K.Ö., G.S., B.B., Z.A., S.A., completed the experimental phases of the manuscript. P.K. performed all the statistics of the study. All authors read the manuscript and approved its submission.

## Conflict of Interest

The authors disclose no potential conflicts of interest.

## Data Availability

Data available on request from the authors.
